# Effects of varying illumination on ocular aberrations and aberration compensation before and after small incision Lenticule extraction: a prospective cohort study

**DOI:** 10.1186/s12886-021-02084-1

**Published:** 2021-09-16

**Authors:** Weiting Hao, Yan Wang, Tong Cui, Wenxing Ning, Qing Zhu, Yaohua Zhang

**Affiliations:** 1 Department of Ophthalmology, The second Hospital of Tianjin Medical University, No. 23 Pingjiang Road, He-xi District, Tianjin, 300211 China; 2 Tianjin Eye Hospital, Tianjin Eye Institute, Tianjin Key Laboratory of Ophthalmology and Visual Science, No. 4 Gansu Road, He-ping District, Tianjin, 300020 China

**Keywords:** Small incision lenticule extraction, Illumination, Ocular aberration, Aberration compensation

## Abstract

**Background:**

There are few reports regarding the influence of varying illumination on the compensation effect before and after corneal refractive surgery. We aimed to evaluate the changes in refraction, higher-order aberrations, and aberration compensation between mesopic and photopic illumination before and after small incision lenticule extraction.

**Methods:**

In this prospective cohort study, only the right eyes of patients who underwent small incision lenticule extraction for the correction of myopia and myopic astigmatism at the Tianjin Eye Hospital were included. Wavefront refraction and higher-order aberrations were measured preoperatively and 3 months postoperatively under mesopic and photopic illumination. Compensation factors were calculated as 1 − (aberration of the whole eye/aberration of the anterior corneal surface).

**Results:**

Forty patients undergoing small incision lenticule extraction were enrolled. All surgeries were completed without postoperative complications. Preoperatively, the eyes only had a statistically significantly higher (*t* = − 4.589, *p* < .001) spherical refractive error under mesopic vs. photopic illumination (median [interquartile range], − 6.146 [2.356] vs. − 6.030 [2.619] diopters [D]), whereas postoperatively, the eyes also exhibited statistically significantly higher (*t* = − 3.013, *p =* .005) astigmatism (− 0.608 [0.414] vs. − 0.382 [0.319] D). Differences in spherical refraction between the two illuminations were the highest in postoperative eyes (Δ > 0.5 D). Only postoperative eyes exhibited statistically significant elevations (*t* ≥ 4.081, *p <* .001) in higher-order aberrations under mesopic illumination, and only preoperative eyes exhibited statistically significantly enhanced (*χ*^*2*^ = 6.373, *p* = .01 for fourth-order and *χ*^*2*^ = 11.850, *p* = .001 for primary spherical aberrations) and decreased (*χ*^*2*^ = 13.653, *p* = .001 for horizontal trefoil) compensation factors under mesopic illumination.

**Conclusions:**

Exaggerations in higher-order aberrations and myopic shift after small incision lenticule extraction became apparent under mesopic illumination. Slight undercorrection may have an enhanced effect under low illumination and may reduce night vision. The specific changes in compensation effects in preoperative eyes may improve optical quality under mesopic illumination. Postoperative eyes have reduced compensation ability, specifically for spherical aberrations, under mesopic illumination, which may diminish night vision. Further studies that include the measurement of subjective night vision parameters should be conducted.

## Background

Corneal refractive surgery is performed worldwide [1]. Satisfactory vision correction is achieved in most patients; however, certain patients may experience poor night vision even when their general visual acuity is 20/20 or better [2]. The mechanism underlying this phenomenon has not been fully elucidated. Previous studies have revealed that higher-order aberrations (HOAs) may increase after refractive surgery, reporting that they are a potential source of poor night vision [3, 4]. Varying illumination also induces changes in the accommodation of the lens [2, 5], which reduces night vision; therefore, investigation of the effects of varying illumination on optical quality will enhance ophthalmologists’ understanding of postoperative night vision.

Changes in refraction (lower-order aberrations) and HOAs cause degradation in retinal image quality [6–8]. There have been reports of changes in refraction with varying illumination in natural eyes. Leibowitz and Owens [2] discovered that poor night vision may cause a night myopic shift, wherein a person appears to become more nearsighted at low illumination. However, only a few studies have been conducted with the aim of investigating whether night myopia is retained after refractive surgery, or whether varying illumination causes other postoperative changes in refraction. Such studies have generally focused on HOAs. For example, Villa et al. [3] reported that secondary astigmatism, coma, and spherical aberrations increased under night vision conditions after laser in situ keratomileusis (LASIK), and that they were statistically significantly correlated with the halo disturbance index. However, changes in HOAs do not comprehensively explain changes in optical quality.

Furthermore, a compensation effect between the anterior corneal surface and internal ocular optics assists in maintaining stability for optimal optical quality. There is ample evidence for a compensation effect for HOAs in natural eyes [9, 10]. However, corneal refractive surgery may disrupt this effect, leading to higher postoperative aberration values and poorer night vision. Lee et al. [11] reported that, compared with preoperative aberrations, postoperative anterior corneal aberrations exceeded the compensation effect of the internal ocular optics, resulting in a statistically significant increase in overall ocular aberrations. Nevertheless, there are few reports describing the influence of varying illumination on the compensation effect before and after corneal refractive surgery. Such studies may aid in elucidating the mechanism of changes in optical quality under different levels of illumination.

The objective of our current study was to investigate the differences in refraction, HOAs, and compensation for aberrations between mesopic and photopic illumination before and after small incision lenticule extraction (SMILE). To the best of our knowledge, no similar study has been reported to date, and this study may contribute to a better understanding of postoperative night vision after SMILE.

## Methods

### Aim

The objective of our current study was to investigate the differences in refraction, HOAs, and compensation for aberrations between mesopic and photopic illumination before and after SMILE.

### Participants

This prospective study involved 40 consecutive participants (the right eye of each patient was selected) who underwent SMILE at Tianjin Eye Hospital, Tianjin Medical University, China, for the correction of myopia and myopic astigmatism between June and November 2016. The inclusion criteria were as follows: age ≥ 18 years, stable refraction (a change ≤0.50 diopters [D]) in the past 2 years, discontinuation of soft contact lens use ≥2 weeks and of rigid gas permeable lens use ≥4 weeks, a clear cornea without opacities, central corneal thickness > 500 μm, residual stromal bed thickness > 250 μm, intraocular pressure < 21 mmHg, and no other ocular conditions. The exclusion criteria were keratoconus (verified or suspected) and systemic diseases such as diabetes or connective tissue disease.

The study protocol adhered to the tenets of the Declaration of Helsinki and was approved by the Ethics Committee of Tianjin Eye Hospital, Tianjin, China (No. 202056). Informed consent was obtained from all participants after thorough explanation of the nature and possible consequences of the procedures were provided.

All participants underwent a thorough preoperative eye examination. Routine examinations included uncorrected distance visual acuity (UDVA) and corrected distance visual acuity (CDVA) examinations, slit lamp microscopy, manifest and cycloplegic refraction, indirect fundoscopy, and tonometry. In addition, wavefront refraction and HOAs were measured both preoperatively and 3 months postoperatively using a wavefront analyzer (KR-1 W, Topcon Corp., Tokyo, Japan). UDVA examination, tonometry, and slit lamp microscopy were also examined postoperatively.

Regarding surgical planning, as the refractive accuracy achieved using manifest refraction is better than that predicted using wavefront refraction [12], the former was used for surgical planning. However, wavefront analyzers can be used to measure refraction at both mesopic and photopic illumination. Additionally, Lebow and Campbell [13] confirmed that spherical and cylindrical refraction measured with a wavefront refractor are suitable for refractive analysis. Therefore, wavefront optometry data were used to analyze differences in refraction between the two illuminations in the current study.

### Measurement of higher-order aberrations

Ocular aberrations included corneal and internal aberrations. Corneal topography was performed, and ocular aberrations measured, using the wavefront analyzer. The device was first used to measure corneal and ocular wavefront aberrations along the same axis; using these measurements as reference points, internal aberrations could be calculated accurately in a relatively short amount of time.

All measurements were performed in a dark room after 10 min of rest, immediately after the participant blinked to reduce tear film-related HOA deterioration. Measurements were conducted for the right eye only, with an undilated pupil, under quiet conditions. Pupil diameter (mm) was used as a reference for defining mesopic and photopic illumination, as there is an empirical relationship between pupil diameter and illuminance (lx) [14, 15]. Mesopic (0.017 lx) and photopic (10.411 lx) illumination were generated using the wavefront analyzer. Consecutive, automatic measurements of aberrations were performed in triplicate by an experienced technician at each illumination. Real-time aberrations and pupil diameters were recorded for analysis. Readings were considered valid if they adhered to the manufacturer’s guidelines.

### Surgical technique

All procedures were performed by the same experienced surgeon (Y.W.). Three drops of oxybuprocaine hydrochloride (Benoxil) were applied 3 min before surgery for topical anesthesia. Thereafter, participants were instructed to fixate on a target light to allow the initiation of suction. The surgeon confirmed the alignment of the center of the ablation zone with the center of the pupil. Thereafter, surgery was performed using a 500 kHz VisuMax femtosecond laser (Carl Zeiss Meditec AG, Jena, Germany), with a laser energy of approximately 170 nJ. The details of the surgical procedure have been previously described [16]. Briefly, the laser spots were spaced 1.5 μm apart, creating photodisruption in the stroma. Four cleavage planes were created on the anterior and posterior surfaces of the refractive lenticule on its vertical edge, as well as a single side-cutting incision with a circumference of 2.0–5.0 mm at the 12 o’clock position. Once the femtosecond laser-cutting procedure was completed, the suction was switched off and the refractive lenticule was extracted from the small incision. The diameter of the optical zone was 6.0–6.6 mm, with a transition zone of 0.1 mm. The cap thickness was 110 μm. Nomogram adjustments were implemented for all 40 eyes, set by the same experienced surgeon.

Postoperatively, 0.3% ofloxacin (Tarivid) eye drops were instilled four times daily for 3 days, and 0.1% fluorometholone (Flumetholon) eye drops were instilled four times daily for 2 weeks; the dosages were tapered over 2 months (one drop less every 2 weeks).

### Calculation of the compensation factor

The compensation factor (CF), as defined by Artal and Guirao [17], was calculated as the relative efficiency of the compensation for aberration. In this study, the CF between the anterior corneal surface and the internal ocular optics was calculated as 1 − (*w*/*c*), where *w* was the aberration of the whole eye, and *c* the aberration of the anterior corneal surface. The aberration of the whole eye was equal to the aberration of the anterior corneal surface when CF = 0, i.e., when there was no compensation effect by the internal optics. A compensation effect was present when CF > 0. Typically, the aberration of the anterior corneal surface is partially compensated by that of the internal ocular optics (CF ranging from 0 to 1). Additionally, a negative value (CF < 0) indicates augmentation, indicating that the aberration of the whole eye is larger than that of the anterior corneal surface.

In the current study, which included 40 eyes, the relationship of a certain HOA (*K)* between the internal ocular optics and the anterior corneal surface appeared as a compensation effect in *x* eyes and as an augmentation effect in *y* eyes (*x* + *y* = 40)*.* The proportion of eyes that demonstrated a compensation effect for *K* was calculated as *x*/40 × 100 (ranging from 0 to 100%). A higher value indicates that *K* of the internal ocular optics tends to compensate for *K* of the anterior corneal surface.

### Statistical analysis

All data were collated and calculated using Microsoft Excel 2007 (Microsoft Corp., New Mexico, United States), and statistical analysis was performed using IBM SPSS Statistics for Windows (version 23.0, IBM Corp., New York, United States). Data normality was examined using the Kolmogorov–Smirnov test. Normally distributed data were described as means ± standard deviations, while non-normally distributed data were described as medians [interquartile ranges]. A two-sample paired *t*-test was used for the comparison of wavefront refraction and HOAs between mesopic and photopic illumination. CFs for HOAs were compared between mesopic and photopic illumination using the Chi-squared test. Statistical significance was set as a two-tailed *p-*value < .05.

## Results

Table 1 presents the basic demographics and preoperative clinical data of the 40 participants. The proportion of eyes with a spherical refractive error > 3.00 D (moderate-to-high myopia) was 95.0% (38/40); however, none of the participants had astigmatism > 2.50 D.

**Table 1:** Baseline clinical data and demographics

Parameter	Value
Sex, *N* (%)	
Male	19 (47.5%)
Female	21 (52.5%)
Age (years)	22.2 ± 4.5 (18 to 34)
Preoperative sphere (D)^a^	−5.84 ± 1.70 (− 2.25 to − 9.50)
Preoperative cylinder (D)^a^	−0.95 ± 0.58 (0.00 to − 2.50)
Preoperative manifest spherical equivalent (D)^a^	−6.26 ± 1.76 (−2.63 to −9.88)

Data are presented as the mean ± standard deviation (range) unless otherwise noted.

*D* diopters.

^a^Results from manifest refraction

### Clinical outcomes of small incision lenticule extraction

All surgeries were completed without postoperative complications. At 3 months postoperatively, all 40 (100%) eyes had a UDVA of 20/25 or better. The mean efficacy index at 3 months postoperatively was 0.99 ± 0.11. The CDVA 3 months postoperatively was the same as the preoperative CDVA in 11 (28%) eyes; 16 (40%) eyes gained one line; 10 (25%) gained two or more lines; 3 (8%) lost one line; and none lost two or more lines. The correlation between attempted and achieved refraction was high (correlation coefficient, 0.99). All forty (100%) eyes were within 0.5 D of the attempted refraction, and the astigmatism of all 40 (100%) eyes was within 0.5 D at postoperative month 3.

### Differences in refraction between mesopic and photopic illumination

#### Preoperative differences

The mean preoperative pupil diameter under mesopic and photopic illuminations was 6.90 ± 0.78 and 4.34 ± 0.92 mm, respectively (*t* = 27.392, *p* < .001). As indicated in Table 2, the difference in the preoperative spherical refractive error between mesopic and photopic illumination was significant (*t* = − 4.589, *p* < .001)—natural eyes exhibited a higher spherical refractive error under mesopic illumination than under photopic illumination.

**Table 2:** Pre- and postoperative refraction under mesopic and photopic illumination

Refraction	Mesopic illumination	Photopic illumination	*t*	*p*-value^a^
Preoperative refraction				
Sphere (D)^b^	−6.146 [2.356]	−6.030 [2.619]	−4.589	< .001
Cylinder (D)^b^	−0.911 [1.091]	− 0.943 [0.581]	.952	.347
Postoperative refraction				
Sphere (D)^b^	−1.002 [1.087]	−0.327 [0.542]	−5.853	< .001
Cylinder (D)^b^	−0.608 [0.414]	−0.382 [0.319]	−3.013	.005

Data are presented as the median [interquartile range].

*D* diopters.

^a^Differences between mesopic and photopic illumination; two-sample paired *t*-test

^b^Results from wavefront refraction

#### Postoperative differences

The mean postoperative pupil diameter under mesopic and photopic illumination was 7.01 ± 0.78 and 4.38 ± 0.88 mm, respectively (*t* = 23.084, *p* < .001). As indicated in Table 2, the differences in the postoperative spherical refractive error and astigmatism between mesopic and photopic illumination were significant (*t* = − 5.853, *p* < .001; and *t* = − 3.013, *p* = .005; respectively). Postoperative eyes exhibited a higher spherical refractive error and astigmatism under mesopic illumination than under photopic illumination.

#### Pre- and postoperative differences

The pre- and postoperative differences in refraction between mesopic and photopic illuminations are indicated in Table 3. The postoperative differences in refraction were higher than the preoperative differences, particularly in terms of the spherical refractive error (> 0.5 D). Postoperative eyes exhibited a more obvious enlargement in refraction under mesopic illumination than did preoperative eyes.

**Table 3:** Pre- and postoperative differences in refraction between mesopic and photopic illumination

Refraction	Preoperative differences^a^	Postoperative differences^a^
Sphere (D)^b^	−0.234 [0.345]	−0.676 [0.873]
Cylinder (D)^b^	−0.102 [0.392]	−0.217 [0.435]

Data are presented as the median [interquartile range].

*D* diopters.

^a^The value under mesopic illumination minus the value under photopic illumination

^b^Results from wavefront refraction

### Differences in higher-order aberrations

#### Preoperative differences in third- to sixth-order aberrations

As indicated in Table 4, the differences in preoperative third- to sixth-order aberrations between mesopic and photopic illumination were not significant (*p* > .05)—natural eyes did not exhibit more HOAs under mesopic vs. photopic illumination.

**Table 4:** Pre- and postoperative third- to sixth-order aberrations under mesopic and photopic illumination

HOAs	Mesopic illumination	Photopic illumination	*t*	*p*-value^a^
Preoperative HOAs (μm)				
S3	0.442 [0.333]	0.118 [0.069]	1.300	.201
S4	0.344 [0.280]	0.080 [0.092]	1.352	.184
S5	0.122 [0.103]	0.041 [0.040]	1.277	.209
S6	0.096 [0.090]	0.031 [0.040]	1.478	.148
Total S3-S6	0.593 [0.446]	0.160 [0.137]	1.329	.191
Postoperative HOAs (μm)				
S3	0.658 [0.614]	0.153 [0.163]	4.427	< .001
S4	0.860 [1.053]	0.090 [0.076]	6.277	< .001
S5	0.239 [0.203]	0.040 [0.032]	4.354	< .001
S6	0.276 [0.212]	0.036 [0.025]	7.128	< .001
Total S3-S6	1.201 [1.376]	0.189 [0.197]	5.515	< .001

Data are presented as the median [interquartile range].

*HOA* higher-order aberration, *S3* root mean square of third-order aberrations, *S4* root mean square of fourth-order aberrations, *S5* root mean square of fifth-order aberrations, *S6* root mean square of sixth-order aberrations, *total S3-S6* combined root mean square of third- to sixth-order aberrations.

^a^Differences between HOAs under mesopic and photopic illumination; two-sample paired *t*-test

#### Postoperative differences in third- to sixth-order aberrations

As indicated in Table 4, differences in postoperative third- to sixth-order aberrations between mesopic and photopic illumination were significant (all *p* < .001). Postoperative eyes exhibited more HOAs under mesopic illumination than under photopic illumination.

#### Preoperative differences in spherical aberration, coma, and trefoil

**Fig. 1:**
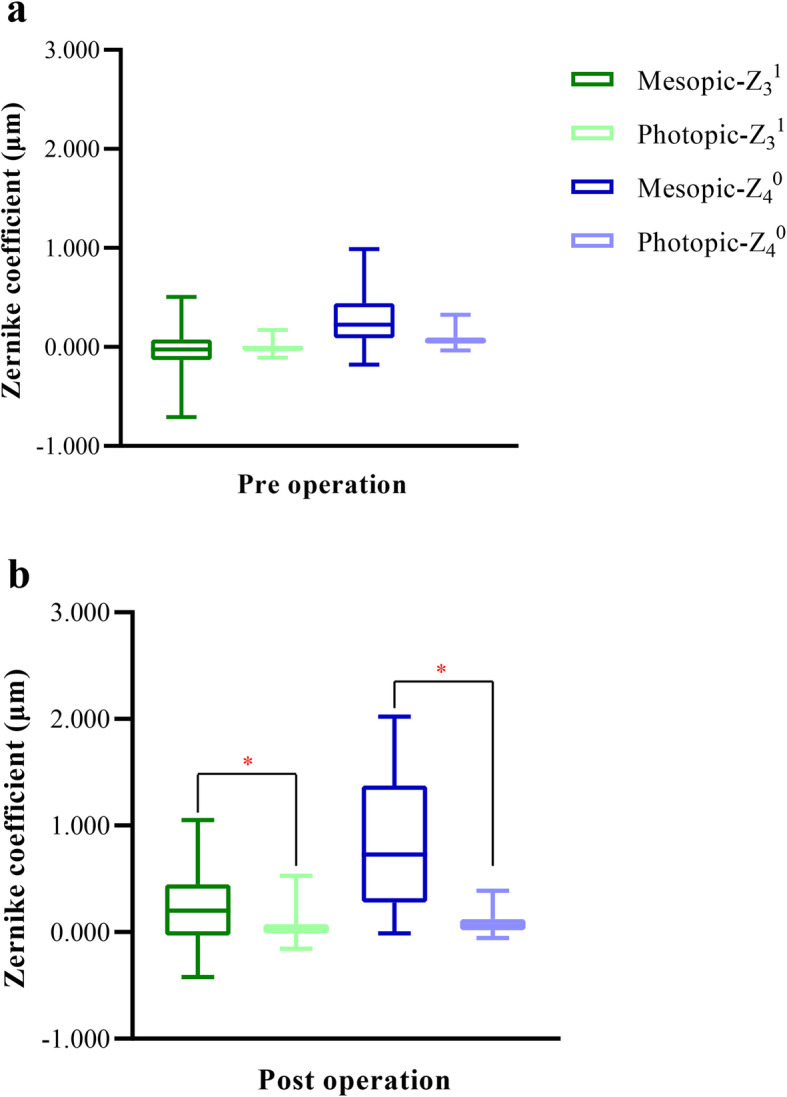
Pre- and postoperative differences in horizontal coma (Z_3_^1^) and primary spherical aberration (Z_4_^0^). (**a**) The preoperative differences in horizontal coma and primary spherical aberration are not significant; two-sample paired *t*-test. (**b**) Postoperatively, the two aberrations are higher under mesopic illumination than under photopic illumination; two-sample paired *t*-test. *Significant difference in aberration values between mesopic and photopic illumination. The upper and lower error bars represent the upper and lower quartiles, respectively. (Z_3_^1^, horizontal coma; Z_4_^0^, primary spherical aberration).

The preoperative differences in Z_4_^0^, Z_3_^− 1^, Z_3_^1^, Z_3_^− 3^, and Z_3_^3^ between mesopic and photopic illumination were not significant (*t* = 1.473, 1.182, −.555, − 1.135, and 1.014, respectively; *p* = .15, .24, .58, .26, and .32, respectively). The preoperative Z_4_^0^ and Z_3_^1^ under mesopic and photopic illumination are illustrated in Fig. 1a.

#### Postoperative differences in spherical aberration, coma, and trefoil

The postoperative differences in Z_3_^− 1^, Z_3_^− 3^, and Z_3_^3^ were not significant (*t* = − 1.209, −.211, and .902, respectively; *p* = .23, .83, and .37, respectively). However, as depicted in Fig. 1b, significant differences in postoperative Z_4_^0^ and Z_3_^1^ were observed between mesopic and photopic illumination (*t* = 6.508, and 4.081; both *p* < .001). Thus, a higher proportion of postoperative eyes exhibited primary spherical aberrations and horizontal coma under mesopic illumination relative to photopic illumination.

### Differences in compensation for higher-order aberrations

#### Preoperative differences in compensation for third- to sixth-order aberrations

**Fig. 2:**
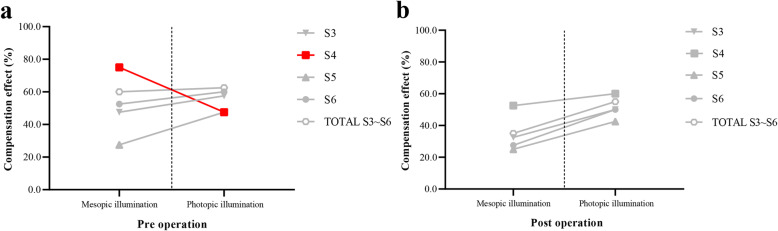
Pre- and postoperative differences in compensation for S3 to S6. (**a**) The proportion of eyes that exhibited a preoperative compensation effect for fourth-order aberrations (red) is significantly higher under mesopic illumination than under photopic illumination; chi-square test. (**b**) The proportion of eyes that exhibited a postoperative compensation effect for third- to sixth-order aberrations does not differ significantly between mesopic and photopic illumination; Chi-squared test. (S3, third-order aberration; S6, sixth-order aberration)

Figure 2 illustrates the proportion of eyes that demonstrated the compensation effect for third- to sixth-order aberrations. A significant difference in the CF distribution between mesopic and photopic illumination was only observed for fourth-order aberrations (*χ*^*2*^ = 6.373, *p* = .01)—natural eyes exhibited a higher compensation effect for fourth-order aberrations under mesopic illumination than under photopic illumination (Fig. 2a).

#### Postoperative differences in compensation for third- to sixth-order aberrations

There were no postoperative differences in the CF distributions for third- to sixth-order aberrations between mesopic and photopic illumination (*χ*^*2*^ = 2.527, .457, 2.739, and 3.413, respectively; *p* = .11, .50, .10, and .07, respectively) (Fig. 2b).

#### Preoperative differences in compensation for spherical aberration, coma, and trefoil

**Fig. 3:**
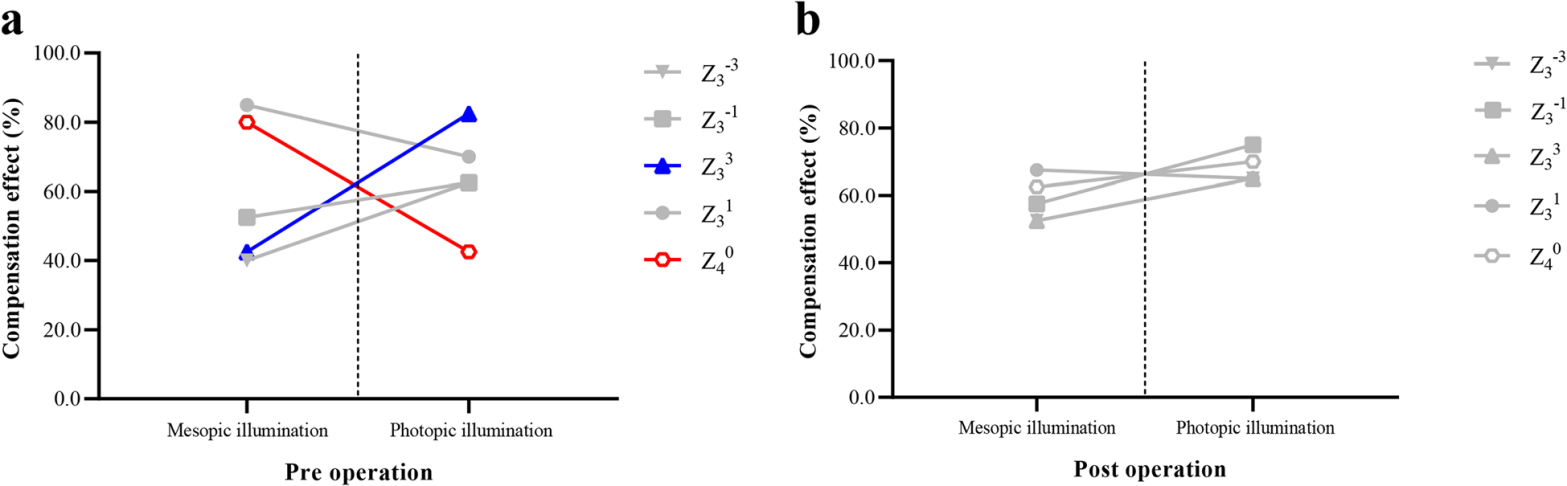
Pre- and postoperative changes in compensation for spherical aberration, coma, and trefoil. (**a**) The proportions of eyes that exhibited a preoperative compensation effect for primary spherical aberration and horizontal trefoil are significantly higher (red) and lower (blue), respectively, under mesopic illumination than under photopic illumination; Chi-squared test. (**b**) The proportion of eyes that exhibited a postoperative compensation effect for spherical aberration, coma, and trefoil does not significantly differ between mesopic and photopic illumination; Chi-squared test. (Z_4_^0^, primary spherical aberration; Z_3_^1^ and Z_3_^− 1^, coma; Z_3_^3^ and Z_3_^− 3^, trefoil)

Figure 3 illustrates the proportion of eyes that demonstrated the compensation effect for primary spherical aberration (Z_4_^0^), coma (Z_3_^− 1^ and Z_3_^1^), and trefoil (Z_3_^− 3^ and Z_3_^3^). Differences were observed in the preoperative CF distributions for Z_4_^0^ and Z_3_^3^ between mesopic and photopic illumination (*χ*^*2*^ = 11.850, and 13.653; *p* = .001, and < .001). The preoperative compensation effect for primary spherical aberration was enhanced, whereas that for horizontal trefoil was reduced under mesopic illumination relative to photopic illumination (Fig. 3a).

#### Postoperative differences in compensation for spherical aberration, coma, and trefoil

There was no difference in the postoperative CF distributions for Z_4_^0^, Z_3_^− 1^, Z_3_^1^, Z_3_^− 3^, or Z_3_^3^ between mesopic and photopic illumination (*χ*^*2*^ = 1.289, 2.739, .056, 1.289, and .503, respectively; *p* = .26, .10, .81, .26, and .48, respectively) (Fig. 3b).

## Discussion

In the current study, the effect of varying illumination on refraction, HOAs, and compensation for aberrations was investigated before and after SMILE.

In previous studies on poor postoperative night vision [18, 19], mathematical conversion was used to compare the differences in HOAs under different pupil diameters. In the current study, the pupil diameter was scaled with varying illumination. Moreover, with the exception of pupil diameter, Leibowitz and Owens [2, 5] reported that accommodation of the lens was different under night vision conditions; therefore, in the current study, we also investigated these changes. Hence, the current study was more representative of real-world conditions than previous studies.

Regarding refraction, we observed a night myopic shift in both natural and postoperative eyes. In addition, compared with natural eyes, postoperative eyes exhibited an augmentation in astigmatism under mesopic illumination. There are several possible explanations for these findings. First, night myopic shifts caused by aberrations in the lens are not altered by SMILE, as demonstrated in the present study. Additionally, the exaggeration in postoperative positive spherical aberration may affect spherical refractive error [20, 21]. Finally, aberrations such as coma may cause an amplification in astigmatism. Putnam et al. [22] reported that a corrective method that considered interactions between HOAs and lower-order aberrations improved night vision through precise correction of the cylinder.

The greater elevation in refraction under mesopic vs. photopic illumination in postoperative eyes may explain the poor night vision in selected patients. Refraction that is not apparent under photopic illumination may become obvious under mesopic illumination. Bamashmus et al. [23] discovered that uncorrected vision was significantly correlated with the postoperative refraction after LASIK. Thus, the higher refractive error under mesopic illumination may lead to poorer postoperative night vision. Therefore, it is necessary to measure wavefront refraction at different illuminations and correct even small degrees of myopic error—particularly in patients with poor night vision—as these patients may be more sensitive to a myopic shift at low illuminations.

In addition, we observed larger proportions of spherical aberrations and horizontal coma in postoperative eyes, compared with natural eyes, when switching from photopic to mesopic illumination. Similar differences were observed in spherical- and coma-like aberrations between pupils of 3 and 7 mm in diameter after photorefractive keratectomy [24]. These changes may be explained from the perspective of surgical ablation. First, in natural eyes, peripheral corneal flattening and a radial gradient in the refractive index of the lens offset the increase in spherical aberration under low illumination [10]; however, in postoperative eyes, the peripheral cornea is steeper than the central cornea, leading to a higher positive spherical aberration under low illumination. Furthermore, the increase in coma under low illumination is thought to be associated with the presence of mild levels of SMILE-induced decentration [25]. Additionally, there may be a correlation between angle Kappa and coma [26]. Finally, the correction of astigmatism with SMILE creates a lenticule with an oval posterior surface. Hence, the extraction of the oval lenticule from the stroma may be a source of postoperative coma [27].

The observed abundance of HOAs under mesopic relative to photopic illumination also influences postoperative night vision. Chalita et al. [18] evaluated correlations between HOAs and night vision symptoms after LASIK, demonstrating that double vision was correlated with total and horizontal coma, and that starburst was correlated with total coma. In addition, there was a correlation between glare and spherical aberrations. Furthermore, Amigó et al. [21] discovered that differences in spherical aberration were inversely correlated with differences in subjective refraction, using an adaptive optics system. Thus, the higher occurrence of positive spherical aberration under low illumination may result in a myopic shift in postoperative eyes. Additionally, contrast sensitivity worsens with an increase in spherical aberration [28]; therefore, HOA measurements are needed for patients with poor night vision, and wavefront-guided retreatment can be used to improve night vision by reducing induced HOAs, if necessary [29].

There are several reports of compensation effects for aberrations of the anterior corneal surface and the internal optics of natural eyes, which assist in the optimization of optical quality under low illumination [11, 17, 30]. In the current study, we observed stronger compensation effects for fourth-order (75 and 47.5% of eyes demonstrated a compensation effect under mesopic and photopic illumination, respectively) and primary spherical (80 and 42.5% of eyes demonstrated a compensation effect under mesopic and photopic illumination, respectively) aberrations under mesopic illumination in natural eyes. Conversely, a weaker compensation effect for horizontal trefoil (42.5 and 82.5% of eyes demonstrated a compensation effect under mesopic and photopic illumination, respectively) was found under mesopic illumination in natural eyes. Spherical aberrations are one of the most important factors in optical quality. Consequently, this phenomenon may be a mechanism by which natural eyes maintain adequate night vision; however, postoperative eyes seem to lose their spherical aberration-specific compensation ability under low illumination. Therefore, in addition to differences in aberration values, differences in compensation for aberrations may be one reason for poor night vision after SMILE. As stated by Benito, Redondo, and Artal [31], customized procedures should be performed to maintain the natural compensation ability and achieve improved night vision outcomes.

There were some limitations to this study; first, the optical zone was not strictly constrained for all 40 eyes, and may have had an indirect confounding effect on lower- and higher-order aberrations under different illuminations. Second, due to the sample size of this study, stratified analysis could not be conducted based on the level of myopia; additionally, the degree of night myopia varies among individuals. In this study, the changes of refraction under mesopic illumination was represented by an average value. It is necessary to conduct an age-stratified study on a larger sample. Finally, subjective night vision parameters were not included. In future studies, correlations between optical quality parameters (refraction, HOAs, and compensation for aberrations) and subjective night vision parameters need to be investigated, which may help surgeons identify the most important factors affecting postoperative visual quality, as well as determine suitable methods to improve surgical outcomes.

## Conclusions

The results of the current study illustrate that augmentations in both HOAs and myopic shift become apparent under mesopic illumination after SMILE. Therefore, a slight undercorrection, which is easy to ignore in clinical practice, may have an enhanced effect under low illumination and reduce night vision. Finally, postoperative eyes seem to have a low compensation ability, specifically for spherical aberrations, under mesopic illumination, which may be another reason for the deterioration of night vision. These findings may contribute to the improvement of surgical outcomes.

## Data Availability

The datasets generated and analyzed during the current study are not publicly available, as the data also forms part of an ongoing study, but are available from the corresponding author on reasonable request.

## Abbreviations

CDVA: Corrected distance visual acuity
CF: Compensation factor
D: Diopters
HOA: Higher-order aberration
LASIK: Laser in situ keratomileusis
lx: Illuminance
SMILE: Small incision lenticule extraction
UDVA: Uncorrected distance visual acuity
